# A randomized, double‐blind, placebo‐controlled phase 1 and phase 2 clinical trial to evaluate efficacy and safety of a SARS‐CoV‐2 vaccine SCoK in adults

**DOI:** 10.1002/ctm2.1016

**Published:** 2022-09-14

**Authors:** Deyan Luo, Hongxing Pan, Peng He, Xiaolan Yang, Tao Li, Nianzhi Ning, Xin Fang, Wenjing Yu, Mingwei Wei, Hui Gao, Xin Wang, Hongjing Gu, Maodong Mei, Xinwang Li, Liangyan Zhang, Deyu Li, Chunrun Gao, Jinbang Gao, Guoqiang Fei, Ying Li, Yuguo Yang, Yi Xu, Wenjin Wei, Yansong Sun, Fengcai Zhu, Zhongyu Hu, Hui Wang

**Affiliations:** ^1^ State Key Laboratory of Pathogen and Biosecurity Beijing Institute of Microbiology and Epidemiology Beijing China; ^2^ NHC Key Laboratory of Enteric Pathogenic Microbiology Jiangsu Provincial Center for Disease Control and Prevention Nanjing China; ^3^ National Institute for Food and Drug Control Beijing China; ^4^ ZHONGYIANKE Biotech Co. LTD Tianjin China; ^5^ Binhai Center for Disease Control and Prevention Yan City China

**Keywords:** clinical trial, safety, SARS‐CoV‐2, vaccine SCoK

## Abstract

**Background:**

To determine an appropriate dose of, and immunization schedule for, a vaccine SCoK against COVID‐19 for an efficacy study; herein, we conducted randomized controlled trials to assess the immunogenicity and safety of this vaccine in adults.

**Methods:**

These randomized, double‐blind, placebo‐controlled phase 1 and 2 trials of vaccine SCoK were conducted in Binhai District, Yan City, Jiangsu Province, China. Younger and older adult participants in phase 1 and 2 trials were sequentially recruited into different groups to be intramuscularly administered 20 or 40 μg vaccine SCoK or placebo. Participants were enrolled into our phase 1 and 2 studies to receive vaccine or placebo.

**Results:**

No serious vaccine‐related adverse events were observed in either trial. In both trials, local and systemic adverse reactions were absent or mild in most participants. In our phase 1 and 2 studies, the vaccine induced significantly increased neutralizing antibody responses to pseudovirus and live SARS‐CoV‐2. The vaccine induced significant neutralizing antibody responses to live SARS‐CoV‐2 on day 14 after the last immunization, with NT50s of 80.45 and 92.46 in participants receiving 20 and 40 μg doses, respectively; the seroconversion rates were 95.83% and 100%. The vaccine SCoK showed a similar safety and immunogenicity profiles in both younger participants and older participants. The vaccine showed better immunogenicity in phase 2 than in phase 1 clinical trial. Additionally, the incidence of adverse reactions decreased significantly in phase 2 clinical trial. The vaccine SCoK was well tolerated and immunogenic.

## Graphical abstract

1. The vaccine SCoK is well tolerated in both younger adults and older adults.

2. The vaccine showed good immunogenicity and broad neutralizing capabilities.

3. A vaccine administered using 40 μg antigen at a three‐dose schedule will undergo an efficacy evaluation in the phase 3 trial.

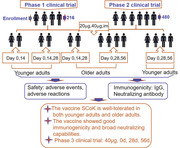

## 1 INTRODUCTION

Severe acute respiratory syndrome coronavirus 2 (SARS‐CoV‐2) is a novel coronavirus that causes coronavirus disease 2019 (COVID‐19).^1^ To date, numerous cases of, and deaths from, SARS‐CoV‐2 are reported daily worldwide. This delta strain of SARS‐CoV‐2 has shown global spread. Omicron has become the world's dominant strain. Whole virus inactivated and mRNA vaccines have already been used widely,^2^, ^3^, ^4^, ^5^ while several human clinical trials are currently underway to assess subunit vaccines and vector vaccine against SARS‐CoV‐2.^6^, ^7^, ^8^, ^9^ Previously, these vaccines showed robust protection from aerosolized SARS‐CoV‐2 in mice and cynomolgus macaques.^10^

The immunogenicity of receptor‐binding domain (RBD) has been verified in animal sera^11^, ^12^ and in samples obtained from patients who had recovered from COVID‐19. These findings suggest that RBD is a promising target for SARS‐CoV‐2 vaccines.^13^ One possible shortcoming of RBD‐based vaccines is that they have lower immunogenicity than full‐length trimeric S protein.^14^ Antibodies play key roles in the protection mediated by RBD‐based subunit vaccines.^15^ However, decreased levels of serum antibody titres induced by monomeric forms of RBD vaccines do not sufficiently predict efficacy in all models.^6^ Therefore, in our previous study, we developed a dimeric form of an RBD vaccine by fusing RBD with the human lgG1 Fc fragment (SCoK); the two RBD domains and the Fc fragment form a Y‐shaped structure.^16^ The Fc fusion protein, which has widely been used as backbone in vaccine development, can be rapidly purified, and increases the half‐life and immunogenicity of target antigens.^17^

After a 2‐year global effort, considerable progress has been made in COVID‐19 vaccine development.^18^, ^19^, ^20^ However, these vaccines still cannot meet the global vaccination demands. In response to the pandemic, we developed a subunit vaccine that is adjuvanted with aluminium hydroxide. While other candidate vaccines currently being evaluated in clinical trials mainly target whole virus or the full‐length spike (S) protein, our vaccine targets the SARS‐CoV‐2 RBD and has shown dramatically enhanced immunogenicity and protection in different animal models.^21^ Herein, we report on pooled analysis of clinical 1/2 trials, in which, vaccine safety and immunogenicity were assessed.

## 2 MATERIALS AND METHODS

### 2.1 Study design and participants

These randomized, double‐blind, placebo‐controlled phase 1 and 2 clinical trials were conducted at the Binhai District, Yan City, Jiangsu Province, China. Trial protocol was reviewed and approved by the National Medical Products Administration, China, and the Institutional Review Board of the Jiangsu Provincial Center for Disease Control and Prevention. This study was performed in accordance with the Declaration of Helsinki and Good Clinical Practice. Eligible participants were healthy adults aged 18 years or older. Individuals who had a history of COVID‐19 or were tested positive for SARS‐CoV‐2 exposure, or who had been in contacted with confirmed COVID‐19 patients were excluded. Eligible older participants were required to be generally healthy and 60 to 85 years of age. General health, assessed during the screening period, was based on clinical laboratory findings, vital signs, physical examination and medical history. Additional exclusion criteria are described in our clinical trial protocol (Protocol file). All the individuals enrolled in this study received this SCoK COVID‐19 vaccine for the first time. In the phase 1 trial, younger (*n* = 144; 18–59 years of age) and older (*n* = 72; ≥ 60 years of age) adult participants were sequentially divided into three distinct groups as follows. In the three‐dose regimen group, participants were randomized to be intramuscularly administered 20 or 40 μg SCoK, or placebo, on days 0, 14, and 28 (allocation ratio, 1:1:1, *n* = 24, 24, and 24 for each regimen, respectively). In the two‐dose regimen group, participants were randomized to be intramuscularly administered 20 or 40 μg of SCoK, or placebo, on days 0 and 14 (allocation ratio, 1:1:1, *n* = 24, 24, and 24 for each regimen, respectively). In the phase 2 trial, younger (*n* = 240; 18–59 years of age) and older (*n* = 240; ≥ 60 years of age) adult participants were sequentially divided into three distinct groups with three‐dose regimen. Participants were randomized to be intramuscularly administered a 20 or 40 μg SCoK, or placebo, on day 0, 28, and 56 (allocation ratio, 5:5:2, *n* = 100, 100, 40 for each regimen, respectively). None of the individual participants were enrolled in both the phase 1 and phase 2 clinical trials. The two trials had different participants.

### 2.2 Randomization and masking

This was a double‐blind, randomized, and placebo‐parallel controlled trial. SAS software (version 9.4) was used to generate a random table of participants in each dose group; participants received an experimental vaccine or placebo labelled with the same number. Individuals performing randomization and masking had no involvement in any other parts of the study. Participants, investigators, and technicians performing laboratory analyses were masked to group allocation.

### 2.3 Procedures

This vaccine SCoK was developed at the Beijing Institute of Microbiology and Epidemiology (Beijing, China) and Zhongyianke Biotech Co. Ltd (Tianjin, China), and contained the RBD of SARS‐CoV‐2/human/CHN/Wuhan_IME‐BJ01/2020 (GenBank number MT291831.1). The vaccine SCoK encoded the SARS‐CoV‐2 RBD antigen (aa331‐524) and human IgG Fc protein as the backbone, which form a dimeric form of RBD. The vaccine was manufactured according to current Good Manufacturing Practice by Zhongyianke Biotech Co. Ltd that was periodically inspected by the National Medical Products Administration committee for compliance. This vaccine is a liquid formulation containing 20 μg or 40 μg per 0.5 ml, with 0.25 mg aluminium hydroxide as the adjuvant each vial. Vaccines were stored at 2 to 8°C before use. Vaccine or placebo was administrated intramuscularly to each participant.

In both trials, participants were monitored for 30 min after vaccination for possible adverse events, and then followed up for any adverse reactions occurring within 7 and 30 days after each dose. All serious adverse events were self‐reported by participants and documented by the investigators. The solicited local adverse reactions included pain, pruritus, rash, redness, and swelling. The solicited systemic adverse reactions included fever, headache, acute allergic reaction, diarrhoea, cough, anorexia, dyspnoea, nausea, vomiting, arthritis, joint pain, fatigue, irritation or inhibition, and mental disorder. Adverse events were graded on the basis of the latest scale issued by China NMPA (Version 2019).

Participants in our phase 1 clinical trial were divided into nine groups and administered two or three doses of the vaccine (at 20 or 40 μg per dose) or placebo on days 0, 14 and on days 0, 14, and 28. Participants in our phase 2 clinical trial were divided into six groups and administered three doses of the vaccine (at 20 or 40 μg per dose) or placebo. Blood samples were then collected from the participants on days 0, 28, 42, and 56 in the phase 1 clinical trial after the first dose. Blood samples were taken from participants in the phase 2 clinical trial on days 0, 70, and 86 after the first dose. An immunoassay (Antobio Diagnostics, Zhengzhou, China) was used to detect specific antibody responses against RBD. Neutralizing antibody responses to a pseudovirus (a vesicular stomatitis pseudovirus expressing parental SARS‐CoV‐2 spike complement) or a live SARS‐CoV‐2 virus (strain SARS‐CoV‐2/human/CHN/Wuhan_IME‐BJ01/2020) were also measured. We also assayed neutralizing antibody responses against other pseudoviruses carrying a spike of delta strain (B.1.617.2), beta strain (B.1.351), or two Omicron strains (B.1.1.529.1 and B.1.1.529.2).

### 2.4 Outcomes

In the phase 1 clinical trial, our primary objective was assessing the safety, and secondary objective was assessing the immunogenicity of the vaccine. In the phase 2 clinical trial, our primary objectives were evaluating the immunogenicity and safety of the vaccine, and determining an immunization schedule and dose for a phase 3 clinical study.

In the safety evaluation, we determined the incidence of adverse events within 30 days of each dose. In the immunogenicity evaluation, we determined the geometric mean titres (GMTs) of RBD‐specific antibody responses, and neutralizing antibody responses against live virus or pseudovirus on day 14 and 28 (or day 30) after the last vaccination. Seroconversion was defined as at least a four‐fold increase in post‐vaccination titre from baseline.

### 2.5 Statistical analyses

The number and ratio of participants with adverse reactions were analysed using Fisher's exact probability method. Antibody levels and neutralizing titres against pseudovirus were calculated using positive proportion and geometric mean titres (GMTs). Analysis of variance (ANOVA) was used to analyse log‐transformed antibody titres. Clopper‐Pearson method was used to calculate 95% confidence intervals (CIs). Fisher exact probability method was used to compare the differences among the differently dosed groups. All statistical analyses were conducted using SAS (version 9.4) and GraphPad Prism (version 8.0.1).

### 2.6 Role of the funding source

Funding agencies were not involved in data collection, statistical analyses, data interpretation, or writing of the manuscript. All the authors had full access to all the data obtained in this study and share the final responsibility for the decision to submit this manuscript for publication.

## 3 RESULTS

### 3.1 Safety assessment

**FIGURE 1:**
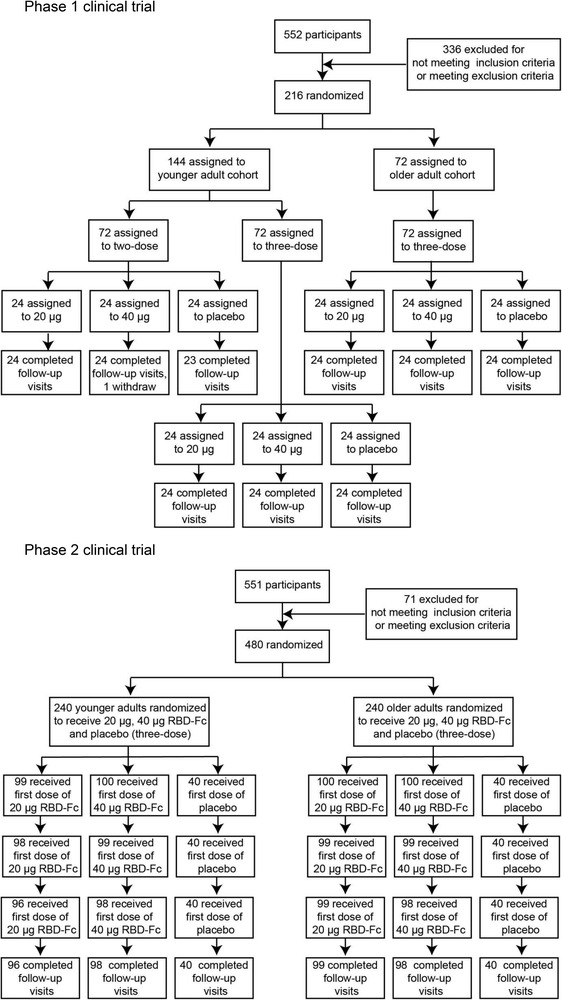
Study protocol for phase 1 and 2 trials. Reasons for exclusion of 10 participants (one in phase 1, and nine in phase 2) from the according‐to‐protocol cohort for vaccination and immunogenicity analysis are listed in Supporting Information 2

In the phase 1 clinical trial, a total of 216 volunteers were enrolled between 1 November 2020 and 1 September 2021 in Jiangsu Province, China. One individual withdrew the consent to participate and left the trial. The remaining participants were randomly divided into nine groups with 24 participants per group. Each group received either vaccine (at 20 or 40 μg dose, SCoK) or placebo (adjuvant‐only). The detailed vaccination schedule and other information are shown in Figure 1 and Table 1. The mean age of younger adult participants was 41.00 years (SD 11.31; range 18–59), with balanced sex and age distribution among the vaccination groups. The mean age of older participants was 66.65 years (SD 3.67; range 60–75). Participants in the two‐dose regimen group received vaccinations on days 0 and 14. Participants in the three‐dose regimen group received vaccinations on days 0, 14, and 28. For the phase 2 clinical trial, a total of 480 volunteers were enrolled between 23 March and 1 September 2021, in Jiangsu, China. Of these, nine individuals withdrew their consent and left the trial. The remaining participants were randomly divided into six groups (*n* = 100 or 40) to receive either vaccine (at 20 or 40 μg, SCoK) or placebo (adjuvant‐only) using a three‐dose schedule, with doses administered 28 days apart. The mean age of the younger adult participants was 44.7 years (SD 10.08; range 18–59), with a balanced age and sex distribution among vaccination groups. The mean age of older participants was 67.02 years (SD 4.81; range 60–82). Each group contained 40 volunteers administered a placebo. All 471 participants in our phase 2 clinical trial completed their full vaccination schedule and follow‐up visits.

**TABLE 1:** Baseline characteristics of the participants classified by age in phase 1 and 2 trial of SCoK

Characteristics	Younger adults (18–59 years)			Older adults (≥60 years)	
	Phase 1 Phase 1		Phase 2	Phase 1	Phase 2
	Two doses	Three doses	Three doses	Three doses	Three doses
Age (years)					
Mean (SD)	40.40 (11.68)	41.6 9 (10.96)	44.70 (10.08)	66.65 (3.67)	67.02 (4.81)
Median	43.00	43.00	47.00	66.00	66.00
Sex(%)					
Male	35 (48.61%)	35 (48.61%)	106 (44.35%)	36 (50.00%)	124 (51.67%)
Female	37 (51.39%)	37 (51.39%)	133 (55.65%)	36 (50.00%)	116 (48.33%)
Ethnicity, *n* (%)					
Han Chinese	72 (100%)	72 (100%)	239 (100%)	72 (100%)	240 (100%)
Other	0	0	0	0	0
High (cm)					
Mean (SD)	164.38 (7.30)	164.31 (8.61)	161.63 (8.01)	161.31 (7.21)	158.74 (7.77)
Median	164.00	164.25	161.50	160.00	159.00
Body weight (kg)					
Mean (SD)	66.89 (11.29)	66.27 (12.54)	68.85 (13.08)	66.26 (8.28)	65.53 (9.95)
Median	65.95	65.30	68.10	66.00	64.85
BMI (kg/m^2^)					
Mean (SD)	24.65 (3.11)	24.43 (3.44)	26.25 (3.98)	25.45 (2.63)	26.01 (3.49)
Median	24.32	24.09	26.08	25.43	25.810

*Note*: Data are mean (SD) or *n* (%).

Within 28 days of each vaccination in the phase 1 clinical trial, 16 (22.22%) of 72 participants in the placebo group, 24 (33.33%) of 72 participants in the 20‐μg dose group, and 26 (36.11%) of 72 participants in the 40‐μg dose group reported at least one solicited adverse reaction. Furthermore, 21 (29.17%) of 72 participants in the placebo group, 30 (41.67%) of 72 participants in the 20‐μg dose group and 30 (41.67%) of 72 participants in the 40‐μg dose group reported adverse events. The most common solicited reaction at the injection site was redness. The most common solicited systemic adverse reactions were cough, fever, and headache. Within 30 days after each vaccination in phase 2 clinical trial, eight (20.00%) of 40 younger participants in the placebo group, 11 (11.11%) of 99 younger participants in the 20‐μg dose group, and 11 (11.00%) of 100 younger participants in the 40‐μg dose group reported at least one solicited adverse reaction; differences among these three groups were not statistically significant (*p* = .313). Moreover, 12 (30.00%) of 40 younger participants in the placebo group, 18 (18.18%) of 99 younger participants in the 20‐μg dose group, and 16 (16.00%) of 100 younger participants in the 40‐μg dose group reported adverse event. None of the differences among these three groups were statistically significant (*p* = .168). The most common solicited reaction at the injection site was pain. The most common solicited systemic adverse reactions were fever, arthralgia, and fatigue. Until 1 September, within 30 days of each vaccination in the phase 2 clinical trial, 2 (5.00%) of 40 older participants in the placebo group, 8 (8.00%) of 100 older participants in the 20‐μg dose group, and 4 (4.00%) of 100 older participants in the 40‐μg dose group reported at least one solicited adverse reaction; differences among these three groups were not statistically significant (*p* = .541). A total of 6 (15.00%) of 40 younger participants in the placebo group, 9 (9.00%) of 100 younger participants in the 20‐μg dose group and 12 (12.00%) of 100 younger participants in the 40‐μg dose group reported adverse events. The difference among these groups were not statistically significant (*p* = .542). The most common solicited reaction at the injection site was pain. The most common solicited systemic adverse reactions were fever, arthralgia, and fatigue (Table 2).

**TABLE 2:** Overall adverse events, solicited local and systemic adverse reactions stratified by age in phase 1 and 2 trial of SCoK

Adverse events/reactions	Younger adults (18–59 years)									Older adults (≥60 years)					
	Phase 1						Phase 2			Phase 1			Phase 2		
	Two doses			Three doses			Three doses			Three dose			Three doses		
	20 μg	40 μg	Placebo	20 μg	40 μg	Placebo	20 μg	40 μg	Placebo	20 μg	40 μg	Placebo	20 μg	40 μg	Placebo
	(*n* = 24)	(*n* = 24)	(*n* = 24)	(*n* = 24)	(*n* = 24)	(*n* = 24)	(*n* = 99)	(*n* = 100)	(*n* = 40)	(*n* = 24)	(*n* = 24)	(*n* = 24)	(*n* = 100)	(*n* = 100)	(*n* = 40)
**Overall adverse events, 28 days for phase 1 clinical trial, 30 days for phase 2 clinical trial**.															
Any	13 (54.17%)	10 (41.67%)	12 (50%)	13 (54.17%)	15 (62.5%)	5 (20.83%)	18 (18.18%)	16 (16.00%)	12 (30.00%)	4 (16.67%)	5 (20.83%)	4 (16.67%)	9 (9.00%)	12 (12.00%)	6 (15.00%)
Vaccination‐related	8 (33.33%)	6 (25.00%)	7 (29.17%)	13 (54.17%)	12 (50.0%)	1 (4.17%)	11 (11.11%)	11 (11.00%)	8 (20.00%)	3 (12.5%)	4(16.67%)	3(12.50%)	8 (8.00%)	4 (4.00%)	2 (5.00%)
Grade ≥3	2 (8.33%)	1 (4.17%)	0	0	4 (16.67%)	0	0	2 (2.00%)	0	0	0	0	0	0	0
**Solicited local adverse reactions 7 days**															
Pain	4 (16.67%)	2 (8.33%)	3 (12.50%)	2 (8.33%)	5 (20.83%)	1 (4.17%)	6 (6.06%)	5 (5.00%)	2 (5.00%)	0	0	0	3 (3.00%)	1 (1.00%)	0
Induration	3 (12.50%)	1 (4.17%)	0	3 (12.50%)	1 (4.17%)	0	0	2 (2.00%)	0	1(4.17%)	1(4.17%)	0	1 (1.00%)	0	0
Swelling	3 (12.50%)	1 (4.17%)	0	4(16.67%)	7(29.17%)	0	3 (3.03%)	1 (1.00%)	0	2(8.33%)	1(4.17%)	0	1 (1.00%)	0	0
Redness	4 (16.67%)	4 (16.67%)	3 (12.50%)	5(20.83%)	5(20.83%)	0	2 (2.02%)	2 (2.00%)	0	2(8.33%)	2(8.33%)	1(4.17%)	1 (1.00%)	0	0
Pruritus	3 (12.50%)	3 (12.50%)	0	9(37.50%)	7(29.17%)	0	1 (1.01%)	1 (1.00%)	0	1(4.17%)	1(4.17%)	0	1 (1.00%)	0	0
**Solicited systemic adverse reactions 7 days**															
Fever	1 (4.17%)	0	0	0	3 (12.50%)	0	6 (6.06%)	4 (4.00%)	6 (15.00%)	1 (4.17%)	0	0	3 (3.00%)	2 (2.00%)	1 (2.50%)
Diarrhoea	0	0	1 (4.17%)	0	0	0	0	0	0	0	1 (4.17%)	0	0	0	0
Arthralgia	0	0	0	0	0	0	0	0	0	0	0	0	0	0	0
Fatigue	0	2 (8.33%)	0	0	1 (4.17%)	0	1 (1.01%)	2 (2.00%)	6 (15.00%)	0	0	0	2 (2.00%)	0	1 (2.50%)
Headache	0	0	0	3 (12.50%)	1 (4.17%)	0	0	0	0	0	0	0	0	0	0
Cough	0	1 (4.17%)	3 (12.5%)	0	0	0	0	0	0	0	1 (4.17%)	1 (4.17%)	0	0	0

*Note*: Data are presented as *n* (%).

### 3.2 Immunogenicity assessment for phase 1 clinical trial

**FIGURE 2:**
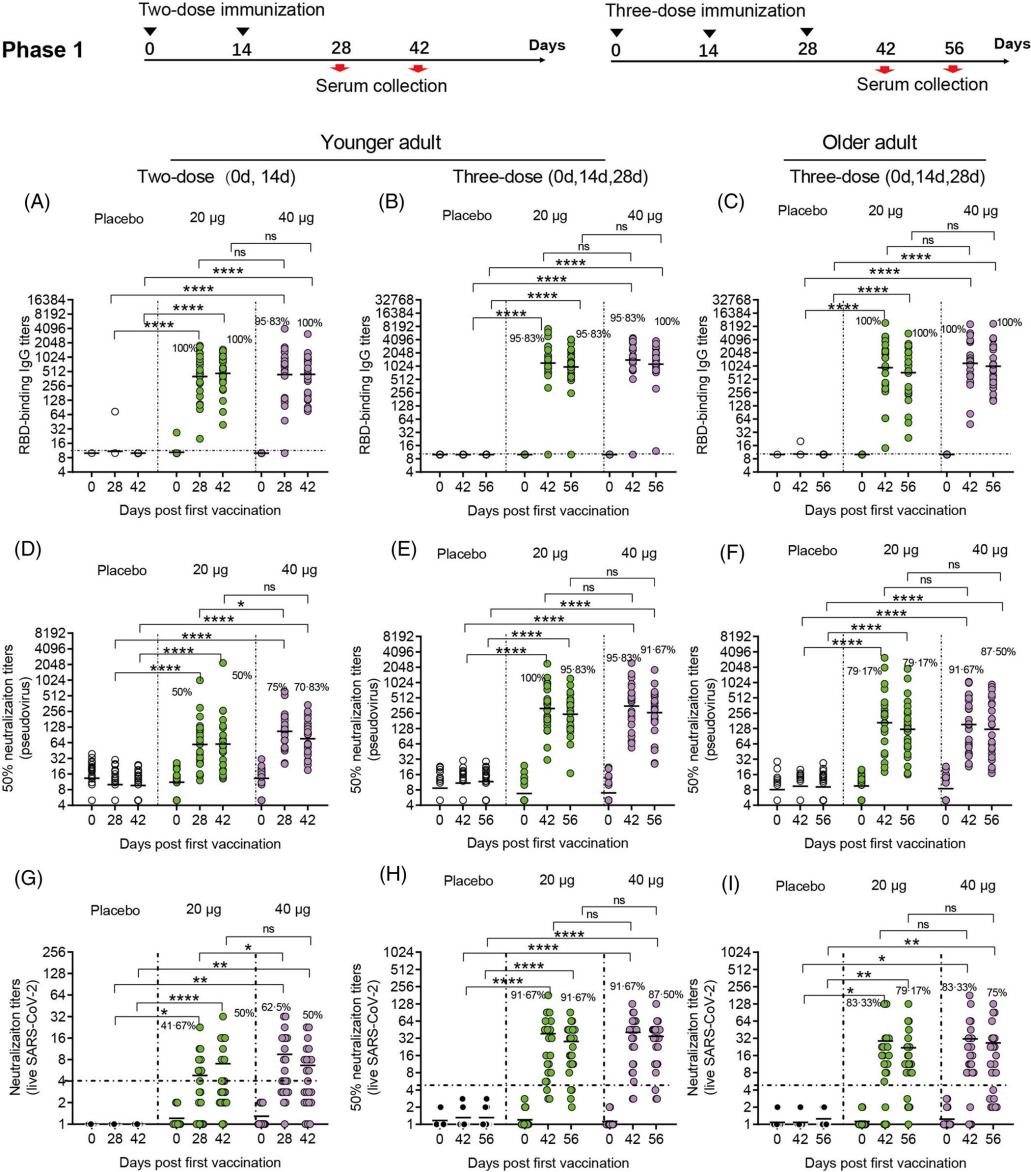
Humoral immune responses in phase 1. Geometric mean titres (GMTs) of RBD binding antibody (A, B, and C), neutralizing antibody titres against pseudovirus (D, E, and F), and neutralizing antibody titres against live virus (G, H, and I) assessed at different time points after the first vaccination are shown. Seroconversion rates are shown at the top of each figure. Horizontal dashed line indicates limit of detection. Analysis of variance (ANOVA) was used to analyse log‐transformed antibody titers.*p < .05, **p < .01, *** *p*< .001, ****p < .0001

**FIGURE 3:**
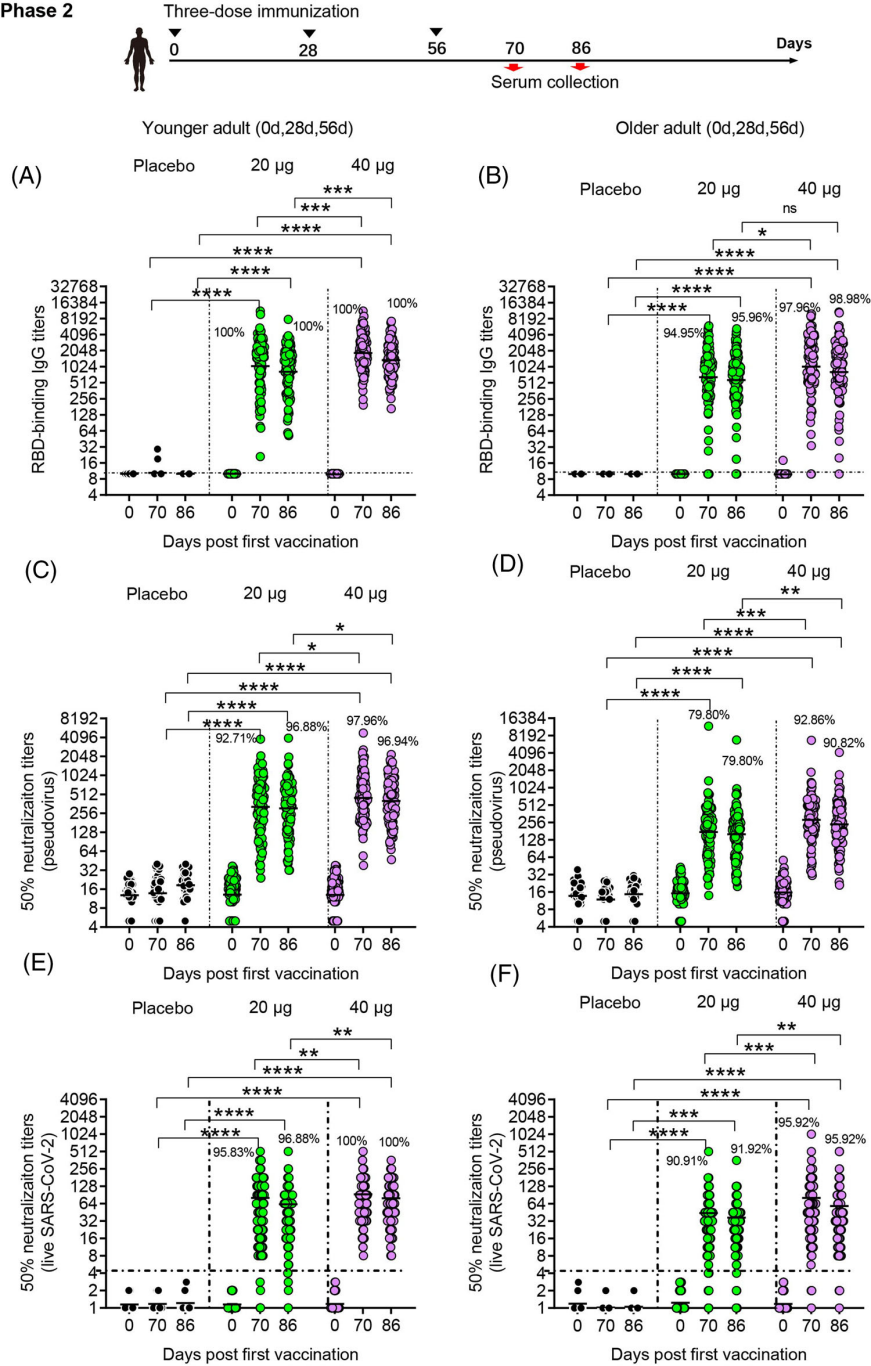
Humoral immune responses in phase 2. GMTs of RBD binding antibody (A and B), neutralizing antibody titres against pseudovirus (C and D), and neutralizing antibody titres against live virus (E and F) assessed at different time points after the first vaccination are shown. Seroconversion rates are shown at the top of each figure. Horizontal dashed line indicates limit of detection. Analysis of variance (ANOVA) was used to analyse log‐transformed antibody titres. *p < .05, **p < .01, ***p < .001, ****p < .0001

To assess immunogenicity in phase 1 and 2 trials, we analysed serologic RBD binding IgG titres and neutralizing antibody titres of all participants (Figures 2 and 3). In younger participants administered two‐dose vaccinations in our phase 1 clinical trial, the antibody responses induced by the vaccine SCoK were detected on day 14 after the last dose, and showed GMTs of 401.81 and 432.29 in the 20 and 40 μg dose groups, respectively. Antibody responses induced by the vaccine SCoK were also detected on day 28, and showed GMTs of 469.34 and 448.13 in the 20 and 40 μg dose groups, respectively. Additionally, 100 and 95.83% had seroconverted at 14 days after the last immunization in the 20‐ and 40‐μg group, respectively; 100% had seroconverted at 28 days after the third immunization in both the 20 and 40 μg groups (Figure 2A). In younger participants administered three‐dose vaccinations, the antibody responses induced by the vaccine SCoK were detected on day 14, and showed GMTs of 1171.89 and 1370.02 in the 20‐ and 40‐μg dose groups, respectively. Antibody responses induced by the vaccine SCoK were also detected on day 28, and showed GMTs of 958.03 and 1139.55 in the 20 and 40 μg dose groups, respectively. Additionally, 95.83% had seroconverted at 14 days after the third immunization in the 20 and 40 μg group, respectively; 95.83 and 100% had seroconverted at 28 days after the third immunization in the 20 and 40 μg group, respectively (Figure 2B). In older participants administered three‐dose vaccinations, the antibody responses induced by the vaccine SCoK were detected on day 14 and showed GMTs of 935.98 and 1178.12 in the 20 and 40 μg dose groups. The antibody responses induced by the vaccine SCoK were also detected on day 28, and showed GMTs of 726.49 and 1018.02 in the 20 and 40 μg dose groups. Additionally, 100% had seroconverted at 14 days after the third immunization in both the 20 and 40 μg group; 100% had seroconverted at 28 days after the third immunization in both the 20 and 40 μg group (Figure 2C). Most of the participants in placebo groups did not show any increase in antibody levels from baseline.

To evaluate neutralizing antibody titres against pseudovirus and live SARS CoV‐2 virus, seroconversion rates and antibody titres were analysed in participants administered a two‐ and three‐dose immunization schedule. In younger participants administered two‐dose vaccinations, both doses induced significantly increased antibody responses to pseudovirus. The high dose induced significantly increased neutralizing antibody responses to pseudovirus, with GMTs of 89.87 and 61.41 detected on days 14 and 28 after the last vaccination, respectively. The low dose also induced significantly increased neutralizing antibody responses to pseudovirus, with GMTs of 60.36 and 58.96 detected on days 14 and 28 after the last vaccination, respectively. Seroconversion of neutralizing antibodies to pseudovirus was 50.00 and 75.00% in the 20 and 40 μg group, respectively, on day 14 after the last vaccination. Seroconversion of neutralizing antibodies to pseudovirus was 50.00 and 70.83% in the 20 and 40 μg group, respectively, on day 28 after the last vaccination. The 50% neutralizing antibody titre in each sample as shown in Figure 2D. In younger participants receiving three‐dose vaccinations, the low doses induced significantly increased antibody responses to pseudovirus, with GMTs of 315.84 and 244.54 detected on days 14 and 28 after the third vaccination, respectively. The high dose induced significantly increased neutralizing antibody responses to pseudovirus, with GMTs of 321.47 and 255.28 detected on days 14 and 28 after the third vaccination, respectively. Seroconversion of neutralizing antibodies to pseudovirus was 100 and 95.83% in the 20 and 40 μg group, respectively, on day 14 after the third vaccination. Seroconversion of neutralizing antibodies to pseudovirus occurred in 95.83% of the 24 participants administered a 20 μg dose, and in 91.67% of the 24 participants administered a 40 μg dose, on day 28 after the third vaccination. The 50% neutralizing antibody titre in each sample are shown in Figure 2E. In older participants administered three‐dose vaccinations, both doses induced significantly increased antibody responses to pseudovirus. The low dose induced significantly increased neutralizing antibody responses to pseudovirus, with GMTs of 163.39 and 121.64 detected on days 14 and 28 after the third vaccination, respectively. The high dose induced significantly increased neutralizing antibody responses to pseudovirus, with GMTs of 143.42 and 125.71 detected on days 14 and 28 after the third vaccination, respectively. Seroconversion of neutralizing antibodies to pseudovirus was 79.17 and 91.67% in the 20 and 40 μg group, respectively, on day 14 after the third vaccination. Seroconversion of neutralizing antibodies to pseudovirus was 79.17 and 87.50% in the 20‐ and 40‐μg group, respectively, on day 28 after the third vaccination. We also assessed the 50% neutralizing antibody titre in each sample as shown in Figure 2F.

In younger participants receiving different dose SCoK vaccinations, both high and low doses induced significantly increased neutralizing antibody responses to live virus. The high dose induced significantly increased neutralizing antibody responses to live virus in younger adults after two‐dose SCoK vaccinations, with NT50s of 9.03 and 6.38 on days 14 and 28 after the last vaccination, respectively. Seroconversion of neutralizing antibodies to live virus was 62.50 and 50% on days 14 and 28 after the last vaccination, respectively (Figure 2G). The high dose induced significantly increased neutralizing antibody responses to live virus in younger adults after three‐dose SCoK vaccinations, showing NT50s of 40.60 and 34.47 on days 14 and 28 after the third vaccination, respectively. Seroconversion of neutralizing antibodies to live virus was 91.67 and 87.50% on day 14 and 28 after the third vaccination, respectively (Figure 2H). The high dose induced significantly increased neutralizing antibody responses to live virus in older adults after three‐dose SCoK vaccinations, with NT50s of 31.12 and 26.64 detected on days 14 and 28 after the third vaccination, respectively (Figure 2H). Seroconversion of neutralizing antibodies to live virus was 83.33 and 75.00%, on day 14 and 28 after the third vaccination, respectively (Figure 2I).

### 3.3 Immunogenicity assessment for phase 2 clinical trial

The immunogenicity of SCoK was also evaluate in phase 2 clinical trial. In younger participants receiving three‐dose vaccinations, the antibody responses induced by the vaccine SCoK were detected on day 14 after the last dose and showed GMTs of 1125.33 (95% CI 883.52–1433.33) and 1843.86 (95% CI 1574.55–2159.24) in the 20 and 40 μg dose groups, respectively. Antibody responses induced by the vaccine SCoK were detected on day 30 after the last dose and showed GMTs of 882.00 (95% CI 710.1–1095.5) and 1356.87 (95% CI 1165.66–1579.45) in the 20‐ and 40‐μg dose groups, respectively (Figure 3A). Additionally, 100% of participants in the two groups had seroconverted at both 14 and 30 days after the third immunization. Most of the participants in the placebo group did not show an antibody increase from baseline. In older participants receiving three‐dose vaccinations, the antibody responses induced by the vaccine SCoK were detected on day 14, and showed GMTs of 636.72 (95% CI 474.14–855.06) and 1099.19 (95% CI 839.71–1438.85) in the 20‐ and 40‐μg dose groups. The antibody responses induced by the vaccine SCoK were detected on day 30 after the last dose, and showed GMTs of 561.96 (95% CI 430.36–733.80) and 941.66 (95% CI 736.95–1203.23) in the 20‐ and 40‐μg dose groups. Additionally, 94.95 and 97.96% had seroconverted at 14 days after the third immunization in the 20‐ and 40‐μg group, respectively. Also, 95.96 and 98.98% had seroconverted at 30 days after the third immunization in the 20 and 40 μg group, respectively. Participants in the placebo group showed no antibody increase from baseline (Figure 3B).

In younger participants receiving three‐dose vaccinations, our vaccine induced significantly increased neutralizing antibody responses to pseudovirus, with GMTs of 321.46 (95% CI 257.12–401.90) and 305.88 (95% CI 249.97–374.30) detected on days 14 and 30 after the third vaccination in the 20 μg group, and GMTs of 447.63 (95% CI 376.75–531.83) and 400.11 (95% CI 337.90–473.78) detected on days 14 and 30 after the third vaccination in the 40 μg group. Additionally, 92.71 and 97.96% had seroconverted at 14 days after the third immunization in the 20 and 40 μg group, respectively. Also, 96.88 and 96.94% had seroconverted at 30 days after the third immunization in the 20 and 40 μg group, respectively. The 50% neutralizing antibody titre in each sample are shown in Figure 3C. In elderly participants receiving three‐dose vaccinations, both doses induced significantly increased neutralizing antibody responses to pseudovirus, with GMTs of 169.23 (95% CI 135.68–211.08) (95% CI 135.68–211.08) and 160.26 (95% CI 132.75–193.47) detected on days 14 and 30 after the third vaccination in the 20 μg group, and those of 282.14 (95% CI 232.76–342.00) and 238.11 (95% CI 198.53–285.60) detected on days 14 and 30 after the third vaccination in the 40 μg group. Additionally, 79.80 and 92.86% had seroconverted at 14 days after the third immunization in the 20 and 40 μg group, respectively. Also, 79.80 and 90.82% had seroconverted at 30 days after the third immunization in the 20 and 40 μg group, respectively. We also assessed the 50% neutralizing antibody titre in each sample as shown in Figure 3D.

In younger participants receiving three‐dose vaccinations, the low dose induced significantly increased neutralizing antibody responses to live virus, with NT50s of 80.45 (95% CI 62.91–97.99) and 62.64 (95% CI 48.87–76.42) detected on days 14 and 30 after the third vaccination, respectively. The high dose induced significantly increased neutralizing antibody responses to live virus, with NT50s of 92.46 (95% CI 75.65–109.27) and 79.28 (95% CI 66.26–92.29) detected on day 14 and 30 after the third vaccination, respectively. Additionally, 95.83 and 100% had seroconverted at 14 days after the third immunization in the 20 and 40 μg group, respectively. Also, 96.88 and 100% had seroconverted at 30 days after the third immunization in the 20 and 40 μg group, respectively (Figure 3E). In older participants receiving three‐dose vaccinations, the low dose induced significantly increased neutralizing antibody responses to live virus, with NT50s of 44.17 (95% CI 32.21‐56.13) and 36.88 (95% CI 28.07‐45.68) detected on days 14 and 30 after the third vaccination, respectively. The high dose induced significantly increased neutralizing antibody responses to live virus, with NT50s of 81.07 (95% CI 55.81–106.34) and 58.34 (95% CI 41.96–74.73) detected on days 14 and 30 after the third vaccination, respectively. Additionally, 90.91 and 95.92% had seroconverted at 14 days after the third immunization in the 20 and 40 μg group, respectively. Also, 91.92 and 95.92% had seroconverted at 30 days after the third immunization in the 20 and 40 μg group, respectively (Figure 3F).

### 3.4 Cross‐notarization against pseudovirus of epidemic strains of SARS‐CoV‐2

We further evaluated whether sera collected after vaccine SCoK vaccination could cross‐neutralize different epidemic strains of SARS‐CoV‐2. Expectedly, vaccinated sera showed increased neutralizing capability against pseudoviruses carried S protein of ancestral virus strain (SARS‐CoV‐2/human/CHN/Wuhan_IME‐BJ01/2020) and delta strain, but scant neutralizing capability against the beta strain^22^, ^23^ and Omicron strains. Together, our results demonstrate that our vaccine induced high levels of antibodies having broad neutralizing capabilities against SARS‐CoV‐2 (Supporting Information 1).

## 4 DISCUSSION

The safety of our vaccine was evaluated in a randomized, double‐blind, placebo‐controlled phase 1 trial. This trial, which was designed using sentinel observation and dose escalation, was conducted in 216 healthy participants recruited from young adult and geriatric population, and was followed by a phase 2 trial. We found that vaccination using 20 or 40 μg doses, and a two‐ or three‐dose schedule, was well‐tolerated in our study cohort. When we adjusted our immunization program to a new schedule (0 day, 28 days, 56 days) in phase 2 clinical trial, the safety profile of the vaccine was improved. There were no differences in the incidence of adverse reactions between the vaccine and placebo groups. Most adverse reactions were mild with most common ones being injection‐site pain, redness, and swelling. Occurrences of fever and fatigue were rare. Expectedly, vaccine SCoK showed an acceptable safety profile in both phase 1 and 2 trials, even in groups having elderly participants. The vaccine SCoK was developed by fusing the RBD of the virus with human IgG1Fc. Considering the safety of vaccine, we continued to evaluate the anti‐Fc antibody titres in participants’ samples. The anti‐Fc antibody cannot be detected (data not shown). If the vaccine were to be used on a global scale, immunity towards this region could be a significant safety concern. Thus, we will keep on evaluating the development of antibodies towards Fc domain in phase 3 clinical trial.

We used a microcytopathic effect assay to determine neutralizing antibody titres in serum samples collected from vaccinated participants. Our results indicate that the SCoK vaccine elicited significant humoral responses against COVID‐19. The three‐dose vaccination schedule dramatically enhanced antibody responses compared with those induced by a two‐dose vaccination schedule. Increasing the antigen dose from 20 to 40 μg improved the immunogenicity of the vaccine. The vaccination schedule of every 2 weeks may have dampened the immune responses because this approach may not have allowed appropriate memory reposes to form. Therefore, a vaccine administered using 40 μg antigen at a three‐dose schedule (at day 0, 28, and 56) will undergo an efficacy evaluation in phase 3 trials. The vaccine SCoK showed improved immunogenicity in phase 2 trial after immunization programme schedule was adjusted to a three‐dose regimen (days 0, 28, and 56). Notably, immunogenicity assessed using NT50s and anti‐RBD IgG titres differed between the lower and higher dose regimens. Humoral responses provide protection against SARS‐CoV‐2.^24^, ^25^ The safety and immunogenicity data from our phase 1 and 2 clinical trials assessing this vaccine support the use of high dose administered using a three‐dose schedule, which will undergo a large‐scale evaluation for safety and efficacy in the ongoing phase 3 trial.

Other clinical studies have shown that increasing age is an independent negative factor impacting vaccine immunogenicity, particularly with respect to neutralizing antibody levels generated in response to a live virus challenge. Nevertheless, our results obtained using immunoassay show that antibodies to RBD, and neutralizing antibodies, assessed at days 14 and 28 or day 30 after the last dose were significantly higher in older vaccine recipients than in older placebo recipients. The safety of our vaccine in older participants was better than that in younger adult participants. Post‐vaccination RBD‐specific antibody and neutralizing antibody responses in older participants administered our vaccine were similar to those of younger adult participants.

These trials had several limitations. First, ethnic diversity in our study was limited to ethnic Han participants. A more diverse range of ethnic backgrounds will be included in our multicentre international phase 3 study. Second, vaccine immunogenicity was assessed in our phase 1 and 2 clinical trials at days 14 and 28 after completing a full vaccination schedule, which did not assess the extended duration of the immune response. Immune responses at later timepoints, including those at least 6 months postvaccination, will be evaluated in a follow‐up investigation. Third, relative to the results reported for some other clinical trials,^6^, ^8^ the neutralization titres seem to wane quite rapidly between day 14 and day 28 after the last vaccine dose. We will continue to test the duration of immunity, and attempt to find the reason for this. Perhaps, we will use another adjuvant.

To evaluate the cross‐reaction against other variants of concern of our vaccine, a panel of serum samples from participants in the phase 2 clinical trial were analysed for their neutralizing antibody titres against pseudovirus. The sera collected in the phase 2 clinical trial showed strong capacity to Delta variants. Most samples retained low but detectable neutralization activity against Omicron, suggesting the immune escape capability of Omicron variants. According to our results and some other reports,^26^, ^27^ we believe some mutations (N440K, E484A) will affect the efficacy of the SCoK vaccine against Omicron VOCs. Nonetheless, our results still showed a cross reaction for these Omicron variants.

In summary, the results obtained in our phase 2 trials confirm that this vaccine SCoK had an acceptable immunogenicity and safety profile. These findings also showed that a 40 μg dose of the SCoK vaccine administered using a three‐dose schedule had good immunogenicity and safety profiles. The upcoming phase 3 efficacy trial will evaluate the administration of 40 μg SCoK vaccine using a three‐dose schedule in younger and older adults.

## CONFLICT OF INTEREST

None of the authors have any competing interests to declare.

## Supporting information

Supporting Information

Supporting Information
