# Supplementary figures and images for: A randomized, double‐blind, placebo‐controlled phase 1 and phase 2 clinical trial to evaluate efficacy and safety of a SARS‐CoV‐2 vaccine SCoK in adults
Source: Clin Transl Med. 2022 Sep 14;12(9):e1016. doi: 10.1002/ctm2.1016 (PMC9473350; doi:10.1002/ctm2.1016)

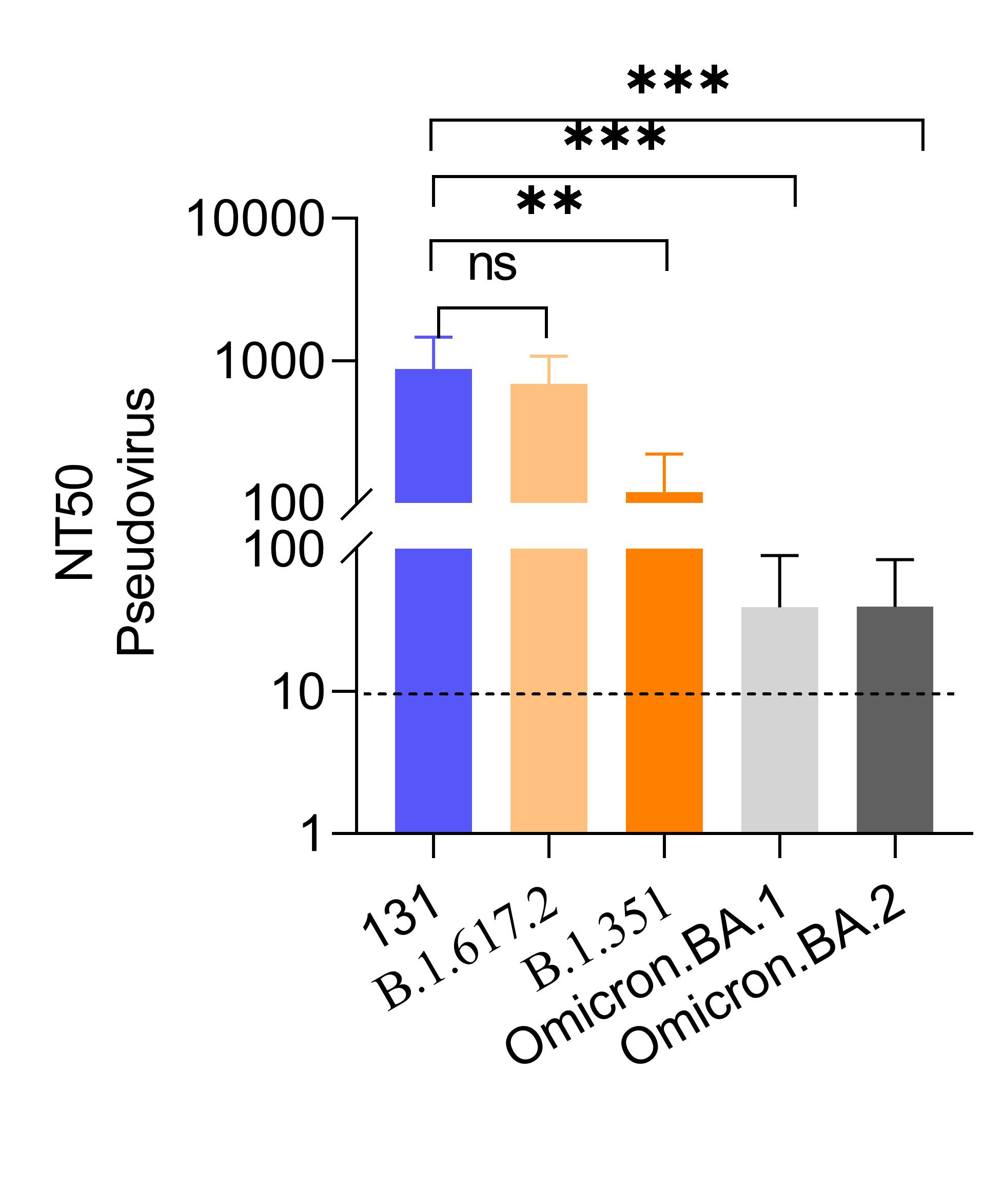

Supplement: Supplementary file 1 — Supporting Information [file CTM2-12-0-s002.jpg]
