# Supplementary material for: A randomized, double‐blind, placebo‐controlled phase 1 and phase 2 clinical trial to evaluate efficacy and safety of a SARS‐CoV‐2 vaccine SCoK in adults
Source: Clin Transl Med. 2022 Sep 14;12(9):e1016. doi: 10.1002/ctm2.1016 (PMC9473350; doi:10.1002/ctm2.1016)
Supplement: Supplementary file 2 — Supporting Information [file CTM2-12-0-s001.docx]

**Supplementary 1**

**Supplementary 2**

| **Number** | **Phase 1** | | **Phase 2** | |
| --- | --- | --- | --- | --- |
|  | **Younger adults** | **Older adults** | **Younger adults** | **Older adults** |
| **Enrolled** | 144 | 72 | 240 | 240 |
| **Completed** | 143(99.31%) | 72(100%) | 234(97.50%) | 237(98.75%) |
| **Withdrew** | 1(0.69%) | 0 | 6(2.50%) | 3(1.25%) |
| **Pregnant** | 0 | 0 | 3(5.00%) | 0 |
| **Withdrew with no reason** | 1(0.69%) | 0 | 3(5.00%) | 3(5.00%) |

**Materials and assay methods**
**Product used in Phase 1/2 clinical trial**The vaccine was manufactured according to current Good Manufacturing Practice by Zhongyianke Biotech Co. LTD., a liquid formulation containing 20 μg or 40 μg per 0.5ml in a vial, with aluminum hydroxide as the adjuvant. The purity of DS (drug substance) was tested using High Pressure Liquid Chromatography and SDS-PAGE. It is 97.81% of batch 20200603A. The stability of DP (drug product) was tested in different conditions. The same batch (20200603A) was used in both phase 1 and phase 2 clinical trials. Before the clinical trials, this vaccine (batch number, 20200502A) was tested in different animal for both efficacy and toxicology, including mouse, rat, guinea pig and monkey. The RBD-ACE2 binding assay was used in vitro. Vaccines were stored at 2℃ to 8℃ before use.

**Evaluation of specific IgG antibodies binding to SARS-CoV-2 RBD**
An immunoassay kits (Antobio Diagnostics CO.,Ltd, Zhengzhou, China) were used to detect the specific antibody responses against the receptor binding domain (RBD). Recombinant human IgG1-Fc protein (103Cys/Ser) was coated to detect anti-Fc antibody titers of samples. Briefly, plates were precoated with RBD or IgG1-Fc protein. Serially diluted sera were added to the plates and incubated for 45 min at 37℃, diluted in 2-fold with a 1:11 dilution as a starting concentration. After four washes, the bound antibodies were detected by incubation with horseradish peroxidase (HRP)-conjugated anti-human IgG antibody for 30 min at 37℃. The reaction was visualized by addition of substrate 3,3’,5,5’-Tetramethylbenzidine (TMB) and stopped by H_2_SO_4_ (1N). The absorbance at 450 nm was measured by a microtiter plate reader (Thermo, SCAN). Value of cutoff was 0.16 plus average value of negative control. IgG titer was the dilution number of endpoints multiplied value of endpoint and divided by value of cutoff. Samples with values ≥ 11 were defined as seroconverted, but those positive on day 0 (Appendix 4) were excluded in the no. of seroconversion.

**SARS-CoV-2 pseudovirus based neutralization assay**We used microcytopathic effect assay to determine neutralizing antibody titers for the serum samples from vaccinees. Sera at 3-fold serial dilutions were incubated with 1024 TCID_50_ of the pseudovirus for 1 hour at 37 °C with a 1:10 dilution as a starting concentration, and then 20000 Huh7 cells were added into each well. DMEM was used as negative control. After 24h incubation at 37℃, the supernatant was then removed and luciferase substrate was added to each well followed by incubation for 2 minutes in darkness at room temperature. Luciferase activity was then measured using GloMax® 96 Microplate Luminometer (Promega). The 50% neutralization titer (NT50) was defined as the serum dilution at which the relative light units (RLUs) were reduced by 50% when compared with the virus control wells. The NT50 was determined by non-linear regression, i.e. log (inhibitor) v.s. normalized response (Variable slope), using GraphPad Prism 8.0 (GraphPad Software). Samples with values ≥ 10 were defined as seroconverted. The pseudoviruses of SARS-CoV-2 strains (BJ01/131, B.1.617.2, B.1.351, B.1.1.529.1 and B.1.1.529.2) were used in this assay

**SARS-CoV-2 wild type virus neutralization assay**
A micro-neutralization assay was carried out to detect neutralizing antibodies against SARS-CoV-2 infection. Briefly, sera at 2-fold serial dilutions were incubated with 100 TCID_50_ SARS-CoV-2( strain BetaCov/human/CHN/Beijing_IMEBJ01/2020, 131), stared 1:2 dilution, delta strain (B.1.617.2，national number: CCPM-B-V-049-2105-8), beta strain (B.1.351, 501Y variant, gene bank number: CSTR.16698.06.NPRC2.062100001) for 1 h at 37℃, and then 50μl (1.5×10^5^/ml) Vero cells were added to each well. The cells were observed daily for the presence or absence of virus-induced Cytopathic Effect (CPE) and recorded at 72 h. Neutralizing antibody titers were determined as the highest dilution of sera that can completely inhibit virus-induced CPE in 50% of the wells (NT50). The baseline of this assay was the 4-fold of assignment. In our study, the value of negative control was assigned to one.

**Evaluation of anti-IgG1Fc antibodies**

Recombinant human IgG1-Fc protein (103Cys/Ser) was coated to detect anti-Fc antibody titers of samples. Briefly, plates were precoated with RBD or IgG1-Fc protein. Serially diluted sera were added to the plates and incubated for 45 min at 37℃, diluted in 2-fold with a 1:11 dilution as a starting concentration. After four washes, the bound antibodies were detected by incubation with horseradish peroxidase (HRP)-conjugated anti-human IgG antibody for 30 min at 37℃. The reaction was visualized by addition of substrate 3,3’,5,5’-Tetramethylbenzidine (TMB) and stopped by H_2_SO_4_ (1N). The absorbance at 450 nm was measured by a microtiter plate reader (Thermo, SCAN). Value of cutoff was 0.16 plus average value of negative control. IgG titer was the dilution number of endpoints multiplied value of endpoint and divided by value of cutoff. Samples with values ≥ 11 were defined as seroconverted, but those positive on day 0 (Appendix 4) were excluded in the no. of seroconversion.

**Statistical analysis**
Statistical analyses were carried out using Prism software (GraphPad). All data were presented as means ± standard error of the means (SEM). Statistical significance among different groups were calculated using the Student’s t test and one-way ANOVA.

**Supplementary 1:** **Cross-neutralizing antibody titers to different pseudoviruses**

The sera of vaccinated participant were assayed the neutralizing antibody responses against pseudoviruses of ancestral virus strain [BJ01(131)], strain B.1.617.2, strain B.1.351 and two Omicron strains (B.1.1.529). The NT50 of each sample was shown, n=13 each group. NS, no significance. **p<0.01.*** p<0.001
